# Probing the Interaction between Nanoparticles and Lipid Membranes by Quartz Crystal Microbalance with Dissipation Monitoring

**DOI:** 10.3389/fchem.2016.00046

**Published:** 2016-12-05

**Authors:** Nariman Yousefi, Nathalie Tufenkji

**Affiliations:** Department of Chemical Engineering, McGill UniversityMontreal, QC, Canada

**Keywords:** nanoparticles, lipid bilayer, quartz crystal microbalance, QCM-D, cell membrane

## Abstract

There is increasing interest in using quartz crystal microbalance with dissipation monitoring (QCM-D) to investigate the interaction of nanoparticles (NPs) with model surfaces. The high sensitivity, ease of use and the ability to monitor interactions in real-time has made it a popular technique for colloid chemists, biologists, bioengineers, and biophysicists. QCM-D has been recently used to probe the interaction of NPs with supported lipid bilayers (SLBs) as model cell membranes. The interaction of NPs with SLBs is highly influenced by the quality of the lipid bilayers. Unlike many surface sensitive techniques, by using QCM-D, the quality of SLBs can be assessed in real-time, hence QCM-D studies on SLB-NP interactions are less prone to the artifacts arising from bilayers that are not well formed. The ease of use and commercial availability of a wide range of sensor surfaces also have made QCM-D a versatile tool for studying NP interactions with lipid bilayers. In this review, we summarize the state-of-the-art on QCM-D based techniques for probing the interactions of NPs with lipid bilayers.

## Introduction

**Figure 1:**
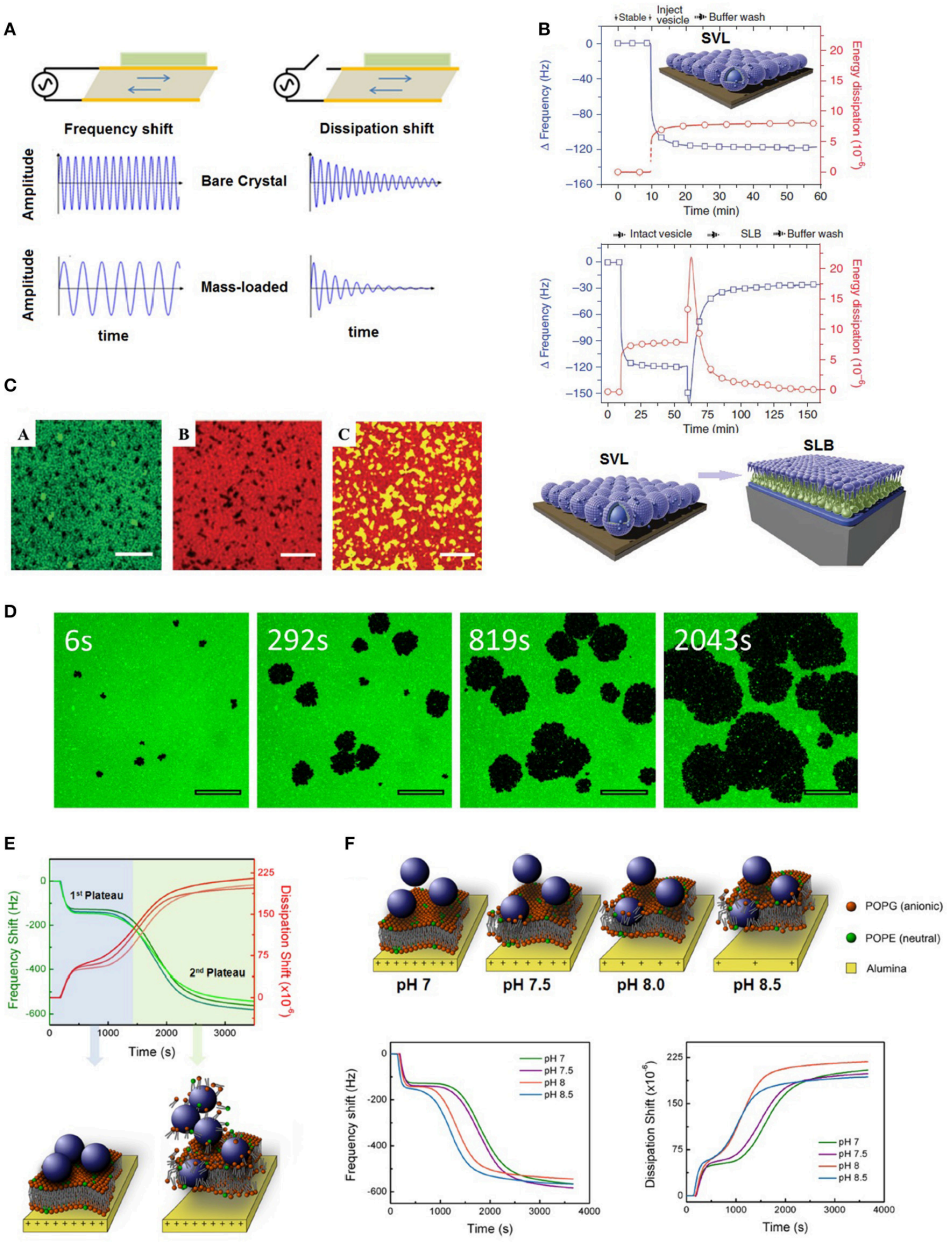
**(A)** Deposition of mass onto a quartz crystal sensor results in changes in its oscillation frequency and dissipation, **(B)** measured QCM-D frequency and dissipation shifts during formation of an SVL and an SLB; Adapted by permission from Macmillan Publishers Ltd: Nature Protocols (Cho et al., 2010), copyright (2010), **(C)** confocal laser scanning microscope image of a lipid raft formed on a QCM-D silica crystal (scale bars represent 2 μm); Adapted from Melby et al. (2016) - Published by the Royal Society of Chemistry, **(D)** pore formation on an SLB by hydrophobic polystyrene NPs (scale bars represent 20 μm); Reprinted with permission from Jing et al. (2014). Copyright (2014) American Chemical Society, **(E)** deposition of positively charged polystyrene NPs on negatively charged SLBs results in a kinetic process of bilayer disruption; Reprinted with permission from Yousefi et al. (2016). Copyright (2016) American Chemical Society, and **(F)** a weaker interfacial interaction between SLBs and their underlying substrates results in faster disruption of bilayers by polystyrene NPs; Reprinted with permission from Yousefi et al. (2016). Copyright (2016) American Chemical Society.

Quartz crystal microbalance with dissipation monitoring (QCM-D) is an acoustic surface sensitive technique for studying phenomena at a wide range of interfaces. QCM-D is essentially a piezoelectric quartz crystal which oscillates at its fundamental frequency in response to an applied AC voltage (Reviakine et al., 2011). As the name suggests, QCM-D is a microbalance in which deposition and detachment of species result in changes in the oscillation frequency (Figure 1A). In addition, when the AC voltage is turned off, the dissipation of energy in the crystal is influenced by the viscoelastic properties and bond stiffness of the adhered species (Kunze et al., 2011). Commercially available QCM-Ds can detect mass depositions as small as a few ng/cm^2^, thus they can be used in detection of small molecules, proteins, viruses, and NPs (Reviakine et al., 2011).

Due to their high specific surface area and enhanced mechanical, optical, electrical, and chemical properties, NPs are being widely used in many engineering applications; however, their environmental fate and transformations, as well as their interaction with cells are subjects of ongoing research. Although, several surface sensitive techniques have been developed for studying the interaction of NPs with model surfaces and membranes, many of them lack the ability to probe their course of interaction in real-time, with molecular level precision and without the use of probe molecules such as dyes. Even in methods such as atomic force microscopy (AFM), the interaction of the probe tip with sample can create experimental artifacts (Picas et al., 2012). QCM-D, on the other hand, provides a label-free and contact-free method for studying the interaction of NPs with model surfaces and supported lipid bilayers (SLBs).

SLBs are planar arrangements of phospholipids that are extensively used as model membranes of mammalian and microbial cells (Chen and Bothun, 2013). Although, mammalian cell membranes are far more complex than SLBs, often containing hundreds of different phospholipids, proteins, and sterols, SLBs replicate their mechanical and barrier properties to a large degree and as such, they can be used for initial probing of the non-specific interactions of NPs with cell membranes (Sackmann, 1996). SLBs are mostly formed by incubation of lipid vesicles with a suitable substrate such as silica or mica, which result in their coalescence and rupture into planar bilayers (Richter et al., 2006). What makes QCM-D an ideal candidate for characterization of SLBs among other surface sensitive techniques, is its sensitivity to differentiate a nearly defect free SLB from a mixture of intact vesicles and SLB patches; while SLBs act like solid films and yield negligible shifts in energy dissipation, vesicles result in much higher dissipation shifts due to their rocking motion in response to crystal oscillations (Richter et al., 2003, 2006). Most optical methods cannot differentiate between SLBs and SLB-vesicle mixtures. In addition, with techniques such as AFM, the interaction of the cantilever tip with any remnant vesicles can result in their disruption (Richter et al., 2006).

The interaction of NPs with SLBs usually starts with their deposition on the bilayers. The deposition of NPs is a phenomenon that can be readily detected using the frequency and dissipation signal of QCM-D (Yousefi et al., 2016). The dissipation to frequency shift ratio (ΔD/Δf) is also a valuable tool which is influenced by the bond stiffness between the adsorbed mass and its underlying substrate (Quevedo et al., 2013, 2014), e.g., the stiffness of the bond between an SLB and the underlying crystal surface. Although, the amount of deposited NPs can be estimated using the frequency shift values, the position and the degree of interaction of NPs with SLBs cannot be resolved by only resorting to the frequency and dissipation shifts. Use of complementary characterization techniques such as fluorescence microscopy, ellipsometry, or AFM is typically necessary to investigate the integrity of the SLBs upon their interaction with NPs. Some of these techniques (e.g., fluorescence microscopy and ellipsometry) can be utilized *in situ* in a QCM-D flow chamber (Olsson et al., 2016), which makes it possible to couple the QCM-D frequency and dissipation data with the complementary method's measurements.

In this mini-review, we will focus on the current advances in using QCM-D for studying NP interaction with SLBs. QCM-D has been also used to probe the interaction of proteins and other biomolecules with SLBs; however, these topics are beyond the scope of this review and we will focus on probing the interaction of NPs with SLBs.

## Formation and characterization of SLBs using QCM-D

Formation of SLBs using QCM-D is typically achieved by the vesicle fusion method. An aqueous dispersion of lipid vesicles is exposed to a chosen substrate through static incubation or continuous flow, which results in the adhesion of vesicles to the substrate. As a result of substrate crowding by neighboring vesicles, they spontaneously collapse to form planar lipid bilayers (Cho et al., 2010). The collapse of vesicles into planar SLBs is accompanied by the release of the vesicular trapped water which results in a positive frequency shift until the final frequency shift is stabilized at approximately –21 to –25 Hz (Richter et al., 2003, 2006; Yousefi et al., 2016). Another significant difference between a supported vesicular layer (SVL) and SLBs is their dissipation shift responses, which is usually <0.2 × 10^−6^ units for SLBs due to their rigid homogeneous nature while vesicles result in much higher dissipation shifts (Richter et al., 2006; Figure 1B).

In addition to simple single component lipids, binary and ternary lipid mixtures have been successfully used for formation of SLBs (Frost et al., 2011, 2012; Karlsson et al., 2013; Yousefi et al., 2016). More realistic SLBs incorporating sterols such as cholesterols (Melby et al., 2016) and lipopolysaccharides (Jacobson et al., 2015) have been successfully formed and characterized using QCM-D. A recent study also reports the formation of phase segregated lipid rafts on a silica coated QCM-D crystal (Melby et al., 2016; Figure 1C).

As mentioned previously, one advantage of QCM-D is the commercial availability of quartz sensors with a wide variety of surface coatings such as gold, silica, alumina, titania, and other metals, metal oxides, and polymers. Coating the sensors with molecules such as l-cystine and self-assembled monolayers (SAMs) has also been reported (Yi and Chen, 2013; Liu and Chen, 2015, 2016). The versatility of surfaces provides opportunities for tailoring the surface interaction of SLBs with their substrates; for example, while gold is electrostatically neutral at physiological pH, silica and alumina carry negative and positive charges, respectively. In addition, surfaces such as alumina and titania are amphoteric; hence, their surface charge can be tuned by changing the medium pH (Yousefi et al., 2016). This principle was recently used in our laboratory for tailoring the interfacial interaction of electrostatically charged SLBs with their underlying substrates (in this case, alumina; Yousefi et al., 2016).

QCM-D can also be used to monitor the phase transition temperature of SLBs (Wargenau and Tufenkji, 2014). It was shown that the gel-to-fluid phase transition of SLBs can be precisely detected as a peak in the derivative of the frequency shift of QCM-D, when the temperature is varied at a constant rate from below to above the transition temperature (or vice versa).

## Probing the interaction of NPs with SLBs using QCM-D

There is growing interest in QCM-D as a valuable method for studying NP interactions with SLBs. Table 1 summarizes the scientific literature on the use of QCM-D for studying SLB-NP interactions.

**Table 1:** **Summary of the literature on probing NP interactions with SLBs using QCM-D**.

**NP type**	**NP size (nm)**	**Lipid type**	**Studied parameter**	**References**
Gold	2, 4, 10, 40	α-PC	Natural organic matter	Bailey et al., 2015
Polyamidoamine	200	POPC:POPS	Drug release	Frost et al., 2011
GO	–	POPC:POEPC	Interaction mechanism	Frost et al., 2012
Gold	4	DOPC+LPS	NP interaction with LPS	Jacobson et al., 2015
Polystyrene latex	28, 62, 140	α-PC	Hydrophobicity and size	Jing and Zhu, 2011
Polystyrene latex	130	α-PC	Ionic type and strength	Jing et al., 2014
Cu, CuO, Cu-Zn	20–200	POPC:POPS	Interaction mechanism	Karlsson et al., 2013
Cu, CuO, Cu-Zn	20–200	POPC:POPG	Interaction mechanism	Karlsson et al., 2013
Polystyrene latex	40, 100	POPC	Hard NP corona	Lesniak et al., 2013
Silica	50	POPC	Hard NP corona	Lesniak et al., 2013
GO	–	DOPC	Ionic type and strength	Liu and Chen, 2015
Silica	36	DOPC	Ionic type and strength	Liu and Chen, 2016
Ceria	39	DOPC	Ionic type and strength	Liu and Chen, 2016
Alumina	38	DOPC	Ionic type and strength	Liu and Chen, 2016
Gold	4	DOPC+Chol.+SM	Lipid raft	Melby et al., 2016
Gold	4	DOPC	Interaction mechanism	Troiano et al., 2014
Gold	4	DOPC:DOTAP	Interaction mechanism	Troiano et al., 2014
Silver	49–65	DOPC	Soft NP corona	Wang et al., 2016
MWCNTs	–	DOPC	Ionic type and strength	Yi and Chen, 2013
CdSe	–	DOPC	pH and ionic strength	Zhang and Yang, 2011
CdSe	–	DOPC:DOTAP	pH and ionic strength	Zhang and Yang, 2011
Airborne particles	<2.5 μm	DOPC, DOPG	Simulated lung fluid	Zhou et al., 2016
Polystyrene latex	20	POPE:POPG	Interfacial interactions	Yousefi et al., 2016

The interaction of a wide variety of NPs such as gold, graphene oxide (GO), multiwalled carbon nanotubes (MWCNTs), silver, polystyrene, silica, metals/metal oxides, and even environmental particulate matter has been studied. The objectives of these studies range from investigating the effect of solution conditions such as pH, ionic strength, and valence on the aggregation and deposition of NPs on SLBs to more complex studies where the effect of substrate-SLB interfacial interactions are investigated. In the following sections, some of these studies are discussed in more detail.

### Effect of solution chemistry on the interactions of NPs with lipid bilayers

Due to the flow-through nature of QCM-D experiments, changing experimental variables such as solution pH, ionic strength, and valence is facile and many of the studies have primarily focused on studying the effect of solution chemistry and other environmental variables on the aggregation and deposition of NPs on SLB coated surfaces (Chen and Bothun, 2013). Yi and Chen (2013) investigated the effect of solution chemistry on the deposition kinetics of carboxylated MWCNTs on zwitterionic 1,2-dioleoyl-*sn*-glycero-3-phosphocholine (DOPC) SLBs. They showed that MWCNTs do not deposit on DOPC in presence of NaCl due to repulsive hydration forces between the carboxyl groups of the nanotubes and the SLB; however, in presence of CaCl_2_, favorable deposition was achieved. The authors related this favorable deposition to the bridging effect of Ca^2+^ ions. They also investigated the interaction of MWCNTs with lipid vesicles by forming SVLs on gold coated QCM-D sensors. By means of cryogenic transmission electron microscopy (cryo-TEM) and SVL studies, they concluded that MWCNT attachment did not compromise the integrity of the lipid bilayers.

In a similar work, Liu and Chen (2015) also investigated the effect of solution chemistry on the interaction of GO, another carbon nanomaterial, with DOPC SLBs and SVLs. They showed that GO deposits on DOPC SLBs in presence of NaCl and CaCl_2_ and similar to MWCNTs, presence of Ca^2+^ ions resulted in a larger mass and faster deposition kinetics due to their bridging effect. Similar to MWCNTs, no disruption of SVLs was detected; however, dye leakage assay proved pore formation in the presence of GO nanosheets. Thus, QCM-D is not sensitive enough to detect small liquid leakage due to reversible pore formation and complementary techniques such as fluorescence microscopy and dye leakage assays are necessary to confirm the integrity of the lipid bilayers.

In an early study, Zhang and Yang (2011) investigated the effect of Ca^2+^ ion and net electric charge of lipids on the interaction of anionic and cationic CdSe quantum dots (QDs). Using QCM-D, they concluded that the interaction of these QDs with SLBs are purely electrostatic in nature and can be controlled by solution chemistry, such as pH and presence of Ca^2+^ ions. One particular observation was that the QDs favorably deposit on SLBs within a pH window where they are electrostatically attractive to each other. They also showed that the range of the favorable pH window can be controlled by the presence of Ca^2+^ ions.

A clearer picture on the effect of various cations and anions on the interaction of NPs with SLBs is given by Jing et al. (2014). They probed the interaction of semi-hydrophobic carboxylated polystyrene latex NPs with zwitterionic L-α-phosphatidylcholine (α-PC) SLBs using QCM-D. In another study by the same researchers, these NPs had been shown to induce pores in α-PC SLBs (Jing and Zhu, 2011). The degree of hole formation was investigated using fluorescence microscopy (Figure 1D). It was concluded that the degree of SLB-NP interactions, as demonstrated by the amount of hole formation by NPs, follows the Hofmeister series (the order by which ions cause protein precipitation from solution) for various anions such as CH_3_COO^−^, Cl^−^, ${\text{NO}}_{3}^{-}$, and SCN^−^, while for cations such as Cs^+^, Rb^+^, Na^+^, $\text{N}{\left({\text{CH}}_{3}\right)}_{4}^{+}$ such order is not observed (Jing et al., 2014).

### Effect of environmental and biological transformations on the interaction of NPs with lipid bilayers

Once they interact with their surrounding environments, most NPs are transformed by processes such as dissolution, adsorption, oxidation, and reduction, occasionally leading to drastic changes of the properties of the “pristine” original NPs. Natural organic matter (NOM)—natural macromolecules that are the by-products of decay—often covers the surface of NPs once they are introduced into aquatic or soil environments (Lowry et al., 2012). In the same fashion, once they enter the body, NPs are covered by proteins, lipids and other biomolecules that are readily available in the bloodstream or extra-cellular matrix to form a corona on the NP surface (Pearson et al., 2014). The environmental and biological transformations of NPs can completely change the NP identity, and hence, its interaction with cell membranes. QCM-D is a powerful tool to probe such interactions and in this section, recent studies on this topic are reviewed.

Bailey et al. (2015) investigated the effect of NOM on the interaction of gold NPs with zwitterionic α-PC SLBs. Polymethylmethacrylate (PMMA) was used as a model NOM. They showed that while bare NPs barely interacted with the bilayers, those coated with PMMA readily deposited on the SLBs, hence they likely may be more cytotoxic in presence of NOM. They also concluded that small PMMA-coated NPs could translocate in the space between the lipid leaflets; however, the QCM-D frequency and dissipation shift data are often not sufficient to express the spatial distribution of NPs and other complementary techniques such as fluorescence microscopy or AFM is required for a confident determination of the location of NPs (Chen and Bothun, 2013).

Recent studies have focused more on the biological transformation of NPs and their effect on their interaction with SLBs. Liu and Chen (2016) investigated the effect of phosphate ions, a prevalent species in biological fluids, on the interaction of silica, ceria and alumina NPs with DOPC SLBs using QCM-D. Although phosphate ions resulted in the favorable attachment of nano-sized silica to SLBs, favorable attachment could not be observed for the other NPs due to phosphate binding to the NP surface.

The effect of protein corona has also been investigated using carboxylated polystyrene latex and unmodified silica NPs (Lesniak et al., 2013). While polystyrene NPs were shown to deposit on 1-palmitoyl-2-oleoyl-*sn*-glycero-3-phosphocholine (POPC) SLBs, coating them with a fetal bovine serum corona resulted in the absence of any interaction with the same SLB. Protein corona is believed to have significantly reduced the surface energy of the polystyrene NPs. Human serum albumin coated silver NPs were also shown to have much lower interaction with DOPC SLBs in contrast to their bare counterparts (Wang et al., 2016). The protein corona was shown to further stabilize the NPs by imparting more negative surface charge and also providing more steric hindrance due to the existence of large protein molecules.

### Advanced lipid nano-engineering: complex multi-component lipid mixtures, NP-lipid assemblies and lipid poration sensors

The versatility of the QCM-D sensor surface provides a considerable advantage as it makes it possible to successfully form complex lipid structures. Incorporation of lipopolysaccharides (LPS) and phase segregated lipid rafts are among these complex structures. Jacobson et al. (2015) investigated the effect of LPS content on the interaction of anionic and cationic gold NPs with POPC SLBs. The outer membrane of Gram negative bacteria contains LPS; hence, incorporating it with POPC will increase the resemblance of the model membrane to that of Gram-negative bacteria. They found that cationic gold NP deposition increased by increasing the LPS content of the bilayer, while this was not the case for anionic gold NPs.

In an effort to better replicate the complex structure of mammalian cells, Melby et al. (2016) formed a lipid raft system incorporating highly ordered domains of sphingomyelin (SM) and cholesterol (Chol.) in a DOPC SLB using QCM-D (Figure 1C). Since the molecular packing and order of these small domains are different from that of the SLB matrix, they often have different responses toward biomolecules and NPs, hence, their role as gateways in cellular membranes. Although, these domains possess the same electrostatic charge as the matrix SLBs, cationic gold NPs were shown by means of QCM-D and AFM to preferentially deposit on the phase segregated domains due to their structural differences from the SLB matrix. The preferential attachment of NPs to the phase segregated domains could be used as a method for rational assembly of NPs into ordered structures, e.g., for targeted delivery applications. The assembly of NPs into a layered structure using QCM-D was also demonstrated for a GO and cationic SLB system for potential sensor applications (Frost et al., 2012).

Controlling and characterizing the interfacial interaction of SLBs with their underlying substrates is a new QCM-D based technique developed in our laboratory (Yousefi et al., 2016). This method is based on the electrostatic interaction between a charged SLB and substrate, whereby the surface charge of the substrate can be tuned by changing medium pH, resulting in controlled electrostatic interaction between the substrate and SLB. The degree of interfacial interaction can be monitored by changes in the QCM-D dissipation signal; a bilayer that is more weakly coupled to the substrate tends to dissipate more oscillatory energy, which is manifested by larger QCM-D dissipation shifts. We also developed a method for probing the integrity of bilayers upon their exposure to cationic and anionic polystyrene latex NPs; while anionic NPs did not impart any damage to the negatively charged SLB, cationic NPs disrupted the bilayers in a kinetic manner (Figure 1E). We showed that the gradual loss of response to pH modulation (that results in reversible changes in the degree of interfacial interactions) could be used as a powerful tool to probe the integrity of SLBs upon their exposure to NPs. This technique paves the way for development of sensors that can monitor bilayer integrity *in situ.* Using this technique, it was also shown that the degree of interfacial interaction between the SLB and its underlying substrate influences the bilayer response toward NPs, where weaker interfacial interactions results in faster disruption of the bilayers (Figure 1F).

## Conclusions and future outlook

QCM-D is a versatile surface sensitive technique for probing the interactions of NPs with model lipid bilayers such as SLBs and SVLs. The sensitivity of QCM-D, along with the possibility to monitor the interaction phenomena *in situ* has made it a technique of choice in studies on NP cytotoxicity, drug delivery and lipid self-assembly. Based on the analysis of the literature, QCM-D has been successfully used to investigate a wide range of lipid systems and NPs, from simple to more complex. However, the role of QCM-D as a sensor for integrity of SLBs has been overlooked and we expect to see more studies on the role of this versatile technique for real-time monitoring of lipid bilayer structural robustness in the near future.

## Author contributions

NY and NT reviewed the literature and wrote the manuscript text.

### Conflict of interest statement

The authors declare that the research was conducted in the absence of any commercial or financial relationships that could be construed as a potential conflict of interest. The reviewer CD and handling Editor declared their shared affiliation, and the handling Editor states that the process nevertheless met the standards of a fair and objective review.

## Nomenclature

α-PC: L-α-phosphatidylcholine
AFM: Atomic force microscope
Chol.: Cholesterol
Cryo-TEM: Cryogenic transmission electron microscope
DOPC: 1,2-dioleoyl-*sn*-glycero-3-phosphocholine
DOPG: 1,2-dioleoyl-*sn*-glycero-3-phospho-(1′-*rac*-glycerol)
DOTAP: 1,2-dioleoyl-3-trimethylammonium-propane
GO: Graphene oxide
LPS: Lipopolysaccharides
MWCNT: Multiwalled carbon nanotube
NOM: Natural organic matter
NP: Nanoparticle
PMMA: Polymethylmethacrylate
POEPC: 1-palmitoyl-2-oleoyl-*sn*-glycero-3-ethylphosphocholine
POPC: 1-palmitoyl-2-oleoyl-*sn*-glycero-3-phosphocholine
POPE: 1-palmitoyl-2-oleoyl-*sn*-glycero-3-phosphoethanolamine
POPG: 1-palmitoyl-2-oleoyl-*sn*-glycero-3-phospho-(1′-*rac*-glycerol)
POPS: 1-palmitoyl-2-oleoyl-*sn*-glycero-3-phospho-L-serine
QCM-D: Quartz crystal microbalance with dissipation monitoring
QD: Quantum dot
SAM: Self assembled monolayer
SLB: Supported lipid bilayer
SM: Sphingomyelin
SVL: Supported vesicular layer
